# Radiocarpal fusion and midcarpal resection interposition arthroplasty: long-term results in severely destroyed rheumatoid wrists

**DOI:** 10.1186/s12891-018-2172-x

**Published:** 2018-08-14

**Authors:** Christoph Biehl, Thomas Braun, Ulrich Thormann, Amir Oda, Gabor Szalay, Stefan Rehart

**Affiliations:** 1 Klinik und Poliklinik für Unfall-, Hand- und Wiederherstellungschirurgie - Operative Notaufnahme, UKGM Gießen, Rudolf-Buchheim-Str. 7, 35392 Giessen, Germany; 2 Klinik für orthopädische Chirurgie der unteren Extremitäten und Endoprothetik, Krankenhaus Rummelsberg GmbH, Rummelsberg 71, 90592, Schwarzenbruck, Germany; 3 Klinik für Orthopädie und Unfallchirurgie, AGAPLESION MARKUS KRANKENHAUS, Chefarzt Prof. Dr. med. Stefan Rehart, Wilhelm–Epstein–Straße 4, D-60431 Frankfurt am Main, Germany

**Keywords:** Rheumatoid arthritis, Partial wrist arthrodesis, Wrist fusion, Rheumatoid wrist, Functional outcome

## Abstract

**Background:**

The aim of this retrospective study is to evaluate distal resection interposition arthroplasty of the wrist as a tool to restore mobility as well as to restore stability in severely destroyed wrist joints.

**Methods:**

Thirty-four wrists in 28 rheumatoid arthritis patients were included. The mean follow-up time was 9 years after surgical treatment with clinical and radiological examination. The results were accessed based on a modification of Clayton ´s scoring system as well as a functional questionnaire.

**Results:**

71% patients were satisfied with pain, function and activities of daily life. Better results were reported by patients with a young age, early surgical intervention, a shorter duration of the disease, and lesser involvement of other joints.

**Conclusions:**

The results for radiocarpal arthrodesis were comparable to those of synovectomy or arthrodesis of the wrist. The results after total wrist joint arthroplasty varies probably as the result of different patient groups, implant types and evolution of prosthetic designs, and are not comparable with the present study.

**Electronic supplementary material:**

The online version of this article (10.1186/s12891-018-2172-x) contains supplementary material, which is available to authorized users.

## Background

Patients with progressive rheumatoid arthritis are severely handicapped in many ways. In more than 90% of patients, the wrist is affected. Any wrist involvement can lead to excessive pain, malfunction and conceivably to a progression of deformation of the fingers, which are usually already compromised in patients with rheumatoid arthritis [1–3]. The wrist is the key joint for an overall treatment strategy for hand and finger deformities and for loss of function [4, 5].

The role of surgical treatment remains a subject of controversy in the clinical management of rheumatoid patients associated with advanced hand dysfunction and destruction (Larsen stage 3–5). In the literature, especially in Anglo-American papers, total wrist arthroplasty (TWA) and total wrist fusion (TWF) appear to be the only solution for rheumatoid wrists, ignoring detailed stage-adapted therapies such as those of the Scandinavian, German and Japanese tradition of rheumatoid surgeons [6–8]. If surgery is indicated in Larsen-stage III-IV, a proximal wrist fusion will ensure stability. In addition, mobilization of the distal row protects limited mobility of the wrist [9].

Correct indication and good clinical results will lead to an alternative to complete arthrodesis or wrist prosthesis. In cases of severely destroyed proximal and distal rows of the carpus, resection interposition arthroplasty (RIAP) provides stability based on radiocarpal fusion, preserving an acceptable range of motion provided by the resection interposition arthroplasty of the midcarpal joint; this, in summary, is advantageous for daily activities [10–12].

In 1981, Tillmann and Thabe modified the proximal resection arthroplasty because of persistent instability to arthrodesis of the proximal row with interposition of the dorsal capsule into the distal joint line for severely destroyed wrists (Larsen IV and V) [13, 14]. The purpose of this retrospective study was to evaluate the clinical and radiological outcomes of 34 distal resection interposition arthroplasty.

## Methods

### Indication

**Fig. 1:**
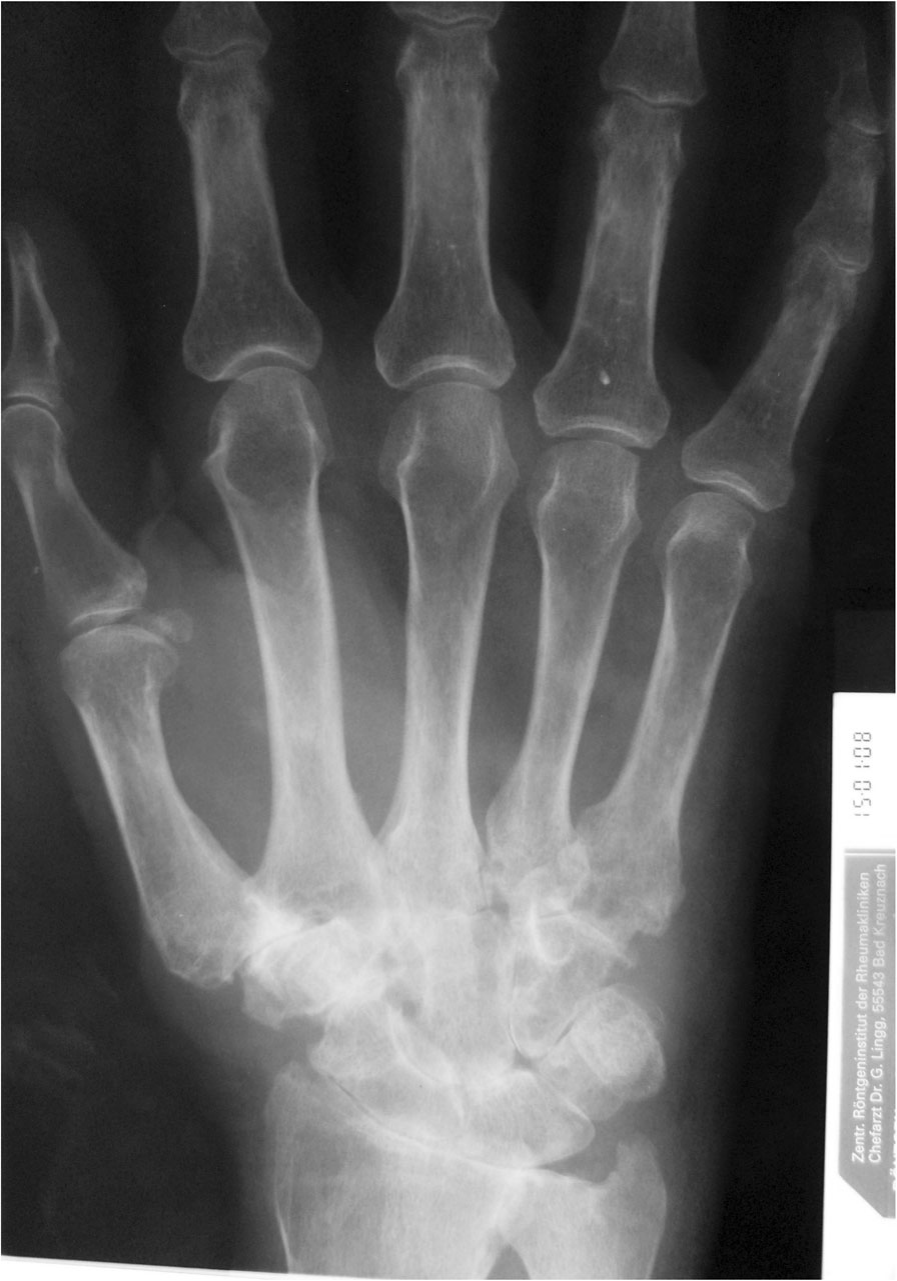
Destructive proximal and distal carpal row in rheumatoid wrist

**Fig. 2:**
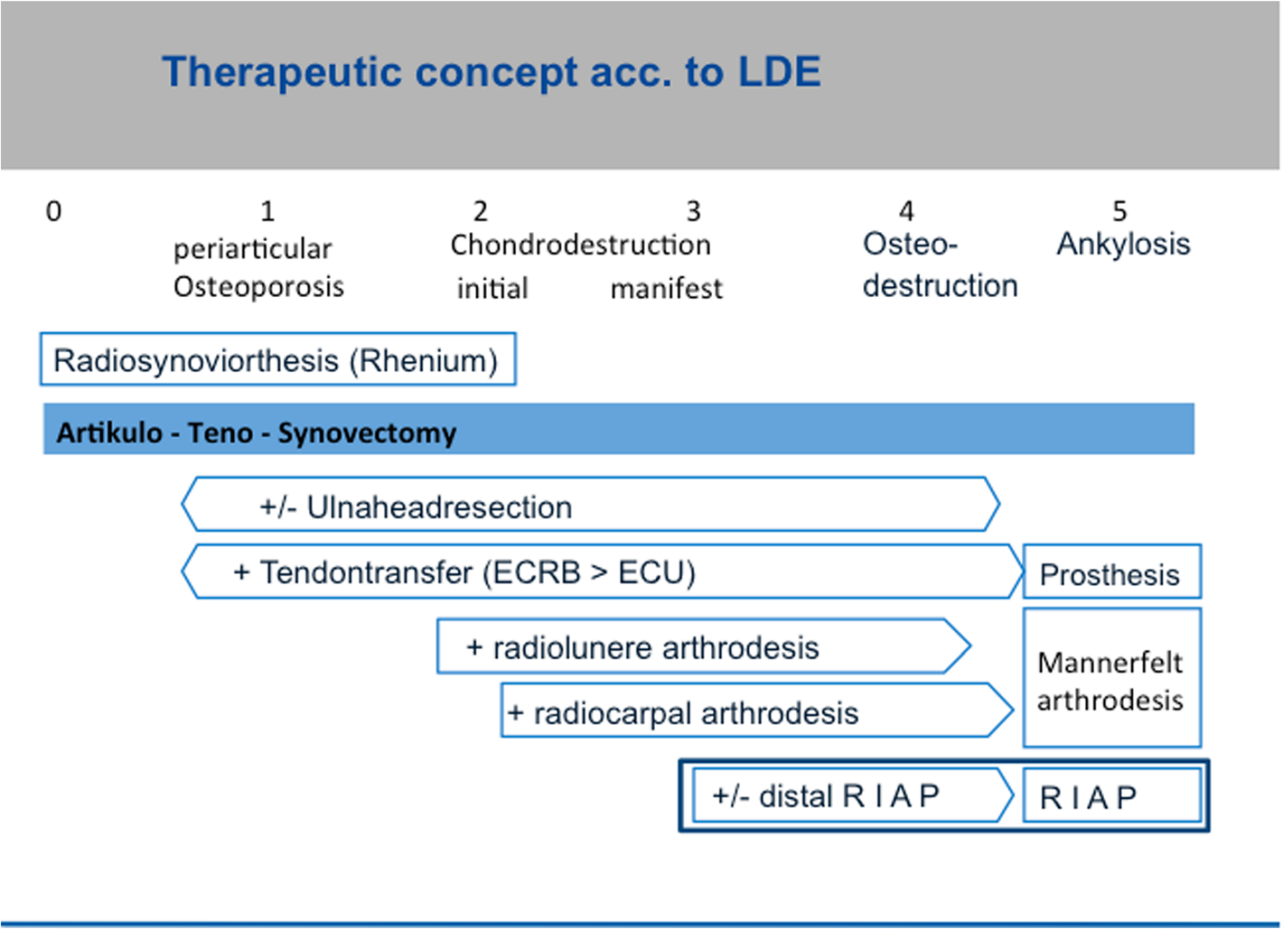
Therapeutic concept according to LDE-classification

In cases of midcarpal joint involvement, according to the progressive stages of Larsen, Dale and Eek (LDE)-classification, total wrist fusion (TWF) was the most common treatment in severely destroyed wrists for more than 70% (Figs. 1 and 2).

At times the decision as to the best surgical treatment was difficult, as in cases of severely destroyed wrist joints in rheumatoid arthritis patients. On the one hand, wrist arthrodesis causes a total loss of flexion and extension, causing difficulties for personal hygiene, the most frequent complaint following arthrodesis. On the other hand, endoprosthetic reconstruction carries the risk of loosening and infection [15]. Neither arthrodesis nor endoprosthetic treatment are completely satisfactory and safe solutions. In an effort to combine the best features of both techniques, radiocarpal fusion can be performed in combination with resection interposition arthroplasty between the lunate and capitate with unacceptable destruction of the capitate head [3, 16].

There are a number of prerequisites for distal resection-interposition-arthroplasty (RIAP) of the wrist joint. The opportunity for this operation depends on destruction-type II or III according to Simmen/Huber, the possibility for soft tissue balancing (tendon transfer), a nearly intact carpal bone height (> 80% of normal high) and an adequate outpatient supply [12, 17]. Four-corner-fusion was the former treatment option, as it was preferred in osteoarthritis patients, but with poor results in rheumatoid arthritic wrists. These were caused by fundamentally different pathologies and primary false transfers from these cases with severe problems of wrist balancing in rheumatoid arthritis patients.

### Surgical procedure [3, 5, 18]

The operation use a dorsal approach. After synovectomy of the extensor tendons denervation of the posterior interosseous nerve is performed, and in cases of ulnar instability, an excision of the distal 2 cm of the ulna is performed.

**Fig. 3:**
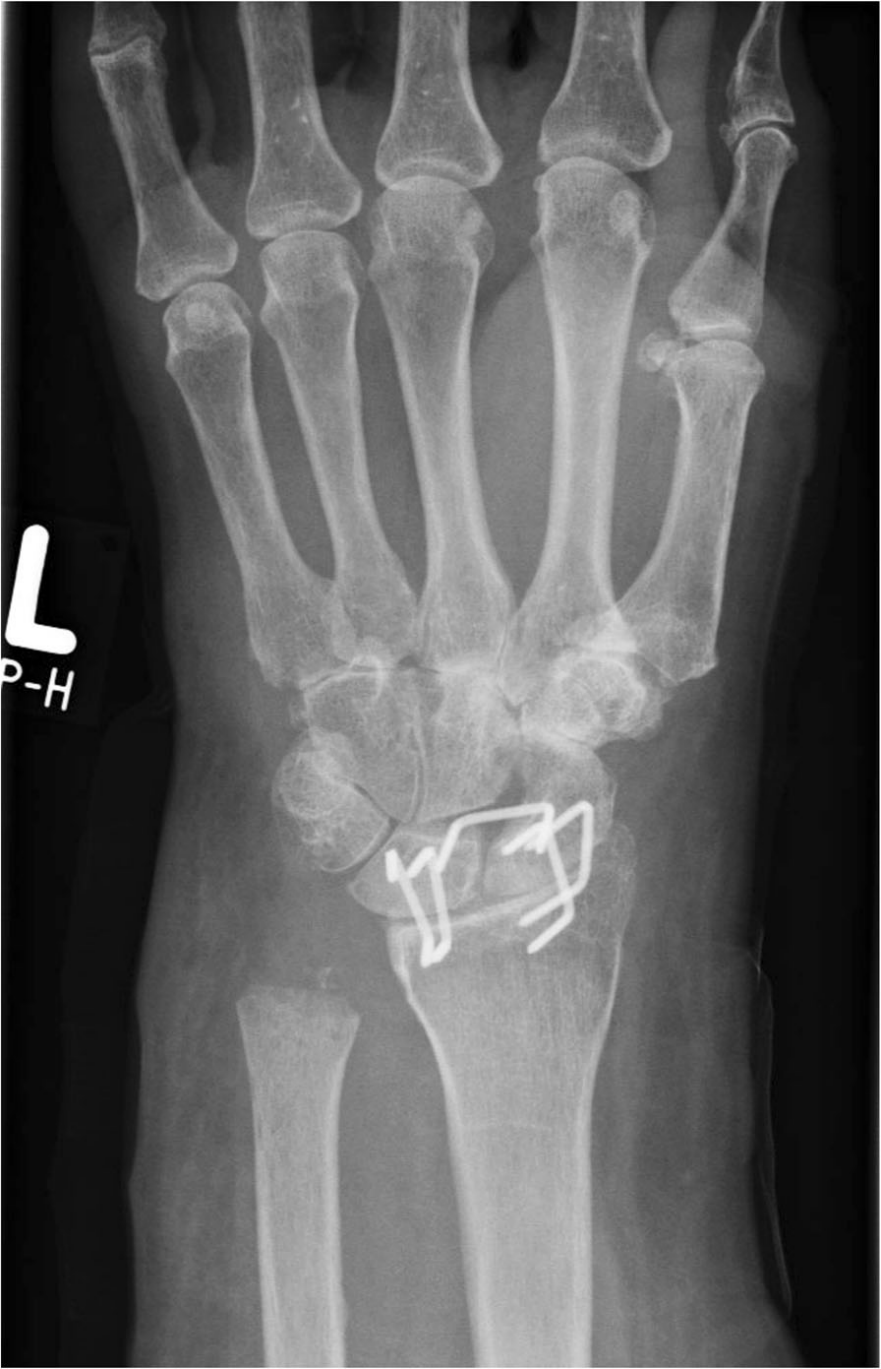
Postoperative X-ray of the wrist after proximal fusion and distal RIAP

The arthrodesis of the proximal row (radiolunate and radioscaphoid) is achieved using staples (Fig. 3) [5, 14], cannulated screws or angular stable plates. Subsequently, approximately 5 mm of the destroyed articular surface of capitate and hamate is resected to rebuild the articular line. A flap of the dorsal capsule or extensor retinaculum is prepared and fixed by interpositioning in the proximal row [5, 18].

Ligament balancing is easier this way for destructive rheumatoid wrists than for carpal arthrodesis, as in four-corner-fusion.

The extensor carpi ulnaris tendon is captured by Swanson’s slope (special prepared retinacula flap) on top of the dorsal ulna [2] or by distal ulna stabilization. Postoperative a special Vainio finger bandage and a volar plaster slab for the wrist should be worn for 6 nights [19]. For more detailed information and figures see the supplementary file “operation technique” (Additional file 1).

### Assessment of the method

Between 1989 and 2002, 28 patients with 34 wrists were treated with distal resection interposition arthroplasty of the wrist at the department of rheumaorthopedic surgery. The average age was 60.6 years. Of these, 78.6% were female, and the dominant hand was affected in 50%. The duration of rheumatoid arthritis ranged from 9 to 54 years with a mean of 18.2 years. The time of follow-up after surgery ranged from 2 to 25 years with an average follow-up of 9.3 years. The interval between the onset of the disease and the operation was an average of approximately 9.1 years.

All patients were examined according to a special protocol. Basic information was recorded, including age, gender, affected side, duration of the disease and date of operation. They were further asked about pain, swelling and certain daily activities. The daily activities were evaluated according to a specially designed questionnaire (modified PROMs) that identified difficulties with common daily activities that could be affected by rheumatoid arthritis (functional questionnaire score, Additional file 2). The score is equal to the Quick-DASH-Score, using the same questions about daily life (Using a knife and fork for eating, hygiene, hair styling, using scissors, elevation of a hat, picking up coins, writing, lying down over the hand, using the keys and opening a bottle). A careful clinical and radiological examination was done to evaluate the degree of joint destruction according to the Larsen-Dale-Eek [20] and Simmen and Huber [17] classifications. The carpal height index and ulnar translation index [21] were also measured.

The results were assessed clinically and radiologically. Radiological examination was routinely performed 6 weeks postoperative. In cases of prolonged bone healing, there was another examination 4 weeks later. In addition, there was another radiological examination if patients reported new or prolonged problems at the time of clinical examination. The clinical assessment was based on a modified Clayton score system [22], as well as a functional questionnaire score, FFbH (4-point Likert-Scale with 30 points for 10 questions) [23]. A score of 30 was the best, and zero was the worst in terms of assessment of patient satisfaction. The clinical score according to the modified 100-point Clayton score was either excellent (90–100 points), good (70–89 points), fair (60–69 points) or poor (< 60 points). At the time of examination of rheumatoid patients in the mid-80s, the DASH-score and other comparable scores had not yet been established. In other studies from this department, we showed that the results of DASH-score and functional questionnaires for activities of daily living were statistically comparable [24].

The statistical analysis of the final results in terms of scores and age, as well as duration from the beginning of the disease to operation were assessed with a two-way ANOVA test and a Mann-Whitney-U-test for non-parametric samples. *P*-values of post hoc tests were adjusted for multiple comparisons. The level of significance was set at < 0.05 for all analyses.

## Results

According to the Clayton score, 10 wrists (29.4%) showed excellent results, 14 wrists (41.2%) were good, 4 wrists (11.8%) were fair, and 6 wrists (17.6%) were poor (Table 1a). The Clayton score includes the parameters balance, mobility, pain reduction and extensor-strength. The fair results were compared to the preoperative data but were devaluated due to handicaps of the other joints. If we combined the categories excellent and good as satisfactory results and the fair plus poor categories as unsatisfactory results, then satisfactory results were found in 24 wrists (70.6%; *p* = 0.00), and unsatisfactory results were found in 10 wrists (29.4%, Additional file 3, Table 1a). The relationship between patient satisfaction and results at follow-up according to Clayton score were statistically significant (Additional file 3, Table 1b). Comparing the Clayton score with the functional questionnaire score, the correlation was also significant.

**Table 1:** a) overall results operated wrists; b) relation between patient satisfaction and functional questionnaire score [27]; c) range of motion and final results at follow-up

	Satisfactory	Unsatisfactory
a) Wrist (Grade)	24 (70.59%)	10 (29.41%)
Excellent	10 (29.4%)	
Good	14 (41.18%)	
Fair		4 (11.76%)
Poor		6 (17.65%)
b) Daily Score		
Minimum – Mean – Maximum	2.00–18.25 – 29.00	0.00–8.30–15.00
Std. Deviation	8.79353	4.73873
t	5.291	
p	.002	
c) Range of motion		
Flexion/Extension	15.97–0 – 22.5	11–0 – 1 .6
Ab−/Adduction	7.2–0 – 13	4.5–0 – 8.7
Pro-/Supination	73.75–0 – 47.9	63–0 – 43.5

The pain details decreases from preoperative 6.3 pts. at the visual analog scale (V A S) to 2.6 pts. at time of follow-up. Most patients changed at time of follow-up from severe pain to mild or no pain. There were differences seen in pain intensity between satisfied patient and these who were not satisfied with the results of the operation. These differences were statistically significant (*p* = 0.058).

Young patients as well as females showed better results, but with no statistical significance. The final results were inversely proportional to the age of the patients. Satisfactory results in young patients (mean age 58 years) were seen in 19 wrists against 15 wrists in older patients (mean age 63.7 years) (*p* = 0.227). From these 24 satisfactory wrists 19 were female wrists (79.2%) and 5 male wrists (20.8%). Unsatisfactory results were measured 6 times in female (60%) and 4 times in male wrists (40%) (*p* = 0.248).

The results were clearer with shorter duration of the disease and with shorter duration between the beginning of the disease and the operation (Additional file 4). This correlation was significant (*p* = 0.049) (Additional file 4). Furthermore, a statistically significant correlation was seen between the time of operation and the final result (*p* = 0.026). In all tests, the correlation was negative and widespread with a Spearman-correlation coefficient of − 0.5.

The operated wrists showed good range of motion especially among patients with satisfactory results (Additional file 3, Table 1c). A slight loss of motion was seen at time of follow-up, even more in patient with unsatisfactory results.

Regarding grip power after operation, we observed better results in 22 wrists, 5 wrists showed no power difference, and 7 wrists had diminished power after the operation. The correlation between wrist power and the final results was statistically significant at follow-up (*p* = 0.028) (Additional file 4). Concurrent problems in shoulder, elbow and fingers negatively affected the power of the wrist joint in these patients. In addition the grip strength was dissected in pinch, weight, extension strength and grip average tests. Except the key pinch all other results showed a positive correlation to the satisfactory subgroup (Additional file 4: Table S7ff).

As part of the score evaluation, patients were examined for shoulder, elbow and finger function. They were asked about pain and problems in daily life and were clinically examined. Patients with mild or no concurrent shoulder, elbow or finger problems showed better final wrist scores than patients with moderate or severe problems. Additionally the subjective results of the upper extremity joints were correlated to different results of the wrists. Next to Clayton-score the relation to grip power were pointed out (Additional file 4: Table S13ff.). Most problems were reported in finger joints in dissatisfied operations (70% severe or poor results with unsatisfactory wrists; *p* = 0.084), followed by shoulder problems (30%; *p* = 0.961) and at last elbow involvement (24%; *p* = 0.566). However, this difference was not statistically significant.

### Radiographic analysis

Hand function, including gripping force, depends primarily on the reconstruction of the carpal height. Only extensive reconstruction permits the physiological bias of the subsequent joints and tendons of the long finger, preventing further destruction or deformity of the carpus. The CHI (carpal height index, standard value: 0.54 +/− 0.03) was used here as a measuring method. In our cohort, this index was 0.4 after reconstruction and bone healing and statistically not significant (*p* = 0.689). This is slightly lower than in that of comparable studies [15: CHI: 0.48]. However, patients were treated surgically with a more advanced LDE stage in our study compared with other studies (LDE IV-V vs. III-IV). This difference was due to the preparation by fusion of the proximal row, and the destruction of the midcarpal row.

The radiological results showed a fusion rate of 94% (32/34 wrists), equal to that of other studies [9, 25].

Two wrists required revision with secondary total arthrodesis after 2 years.

## Discussion

For the operative management of advanced rheumatoid hand destruction, total wrist arthrodesis is the most popular operation for most hand surgeons. Knowledge regarding the rheumatoid wrist, the long-term complex changes in these chronic diseases and the various therapies for these wrists is not widespread [1, 10]. For destroyed wrists of Larsen-stage III to IV, there are some alternative options, including proximal fusion with distal resection interposition arthroplasty. The advantage for total wrist fusion (TWF) is its simplicity, its general predictability and its predictable outcome. Moreover, arthroplasty has a high rate of aseptic loosening and other major complications such as dislocation or progressive instability [15, 26, 27]. For mobility and pain-free activities of daily living, arthroplasty is a highly rewarding procedure in certain conditions. However, operation numbers have decreased in recent years [14].

Most daily activities require combined movement of the wrist at the radiodorsal level, as well as in the ulnopalmar direction [28, 29]. Many kinematic studies have shown that the midcarpal joint is essential for common daily activities [28, 30]. Personal hygiene requires an active extension-flexion arc of 25 degrees, but other activities including eating, drinking, using the telephone or even reading a book require an active range of motion of approximately 40 degrees [16, 28]. Therefore, surgeons need to try to preserve midcarpal function as much as possible [31]. An almost adequate range of motion can be achieved after an appropriately aligned radiolunate or radioscapholunate fusion. Many patients, especially women, prefer the opportunity to have stable joints and acceptable motion, even if it is less [32]. This is because many patients have less pain and become accustomed to the restriction [33]. Tillmann and Thabe also found that natural fusion of carpal joints to an os carpale preserved moderate mobility in combination with less pain [13].

Long-term results of more than 10 years after radiolunate arthrodesis showed sufficient residual mobility [34]. In mutilated wrists, midcarpal instability will persist with collapse of the carpus and lower CHI [30]. These joints could be transferred from an unstable to a stable secondary osteoarthritis form by this procedure [25, 35, 36]. Taleisnik reported that partial arthrodesis/fusion in patients with rheumatoid arthritis was an excellent procedure (up to Larsen III and mild IV) whether performed alone or in association with distal arthroplasty [3]. In our series, the combination of fusion of the proximal row with distal resection interposition arthroplasty offered required stability combined with a sufficient range of motion for coping with most daily activities.

Previous studies by various authors showed that radiocarpal arthrodesis within the first 5 years allowed patients painless movement with a stable proximal joint [3]. In these situations, similar results to those with the isolated proximal arthrodesis could be achieved [3, 37].

In addition to maintaining mobility in the wrist, the achievement of everyday pain relief is of crucial importance. Overall, most studies showed high numbers of pain free wrists (> 85%) with restrictions in joint-projected pain originating mostly from additional affected joints such as elbows or shoulders (34, 36).

Most follow-up studies of TWA report an average mobility of 40° to 50°, comparable with our results [25, 38]. Study results in posttraumatic arthritis were similar to results in rheumatoid patients [39, 40].

Persistent swelling is the main indicator of the activity of rheumatoid arthritis. Synovectomy has a direct impact on the postoperative result, as data have shown [34]. Patients with persistent swelling showed power loss and poor results that were associated with the progression of the rheumatoid disease and not with the operation itself.

Radiocarpal rather than midcarpal fusion is therefore recommended to preserve midcarpal function if the cartilage in the midcarpal joint is intact.

Murphy et al. reported a flexion-extension arc of approximately 76 degrees, a radio-ulnar deviation of 28 degrees and pronation-supination of 168 degrees after wrist arthroplasty, more than we reported in our series after RIAP [27]. Nevertheless, we believe that the stability from radiocarpal arthrodesis combined with a sufficient range of motion from distal RIAP in rheumatoid wrists offers the requirements necessary for the demands of daily activity.

Functional analysis of wrists with arthrodesis of the proximal row showed an oblique plane motion [29]. In addition, the wrist moved almost completely along either the sagittal or coronal plane, as long as forearm function was intact [21]. In affected rheumatoid wrists, the range of motion remained in the same plane but was smaller than in normal wrists.

### Grip power

Stable and pain-free function of the wrist is a prerequisite for postoperatively increasing grip power. In the literature, better grip power, or at least 75% of the normal power in comparison with the opposite side, has been reported [36, 37].

In our series, the postoperative grip power in comparison with the preoperative status increased subjectively: 64% of the wrists had better power postoperatively, and only 20.5% had worse power (*p* = 0.016).

These results were influenced by the progressive destructive nature of rheumatoid arthritis, especially in the fingers and extensor tendons after long-standing rheumatoid disease. In our series, the duration prior to operation ranged from 9 to 54 years. Patients with no or mild finger problems showed significantly better grip power than patients with moderate or severe hand and finger problems.

In our study, we showed satisfactory results in more than 70% of patients after radiocarpal arthrodesis and distal RIAP of the midcarpal joint. In our cases with additional involvement of the midcarpal joint, distal RIAP restored the function of the joint and showed results close to those of an intact midcarpal joint. The mean value of the carpal height index was 0.4, with no significant correlation to the final results according Clayton score. The same applied for the ulnar translation index that had a mean of 0.28 and no significant correlation to the final Clayton score.

In comparison with other studies (also from our department), patient satisfaction showed comparable results to those of patients undergoing synovectomy or total wrist fusion (TWF) [36, 39]. Patient satisfaction with the operation and the outcome depended on finger and grip function [34]. The highest scores were seen in patients with early synovectomy and TWF.

## Conclusion

The distal RIAP [5, 18] is a good alternative treatment for rheumatoid wrists with radiocarpal as well as midcarpal involvement [19, 37], as it provides the stability and satisfactory range of motion that is necessary for daily activities [22]. Revision and conversion to total arthrodesis in cases of unsatisfactory results is a salvage procedure without crucial loss of carpal height. The results of surgical intervention were better in early radiocarpal destructions and we suggest a more active approach than “wait and see” in rheumatic radiocarpal and midcarpal involvement.

## Additional files

Additional file 1: Surgical procedure; operation technique with pictures of intraoperative steps and postoperative treatment (4 pictures). (ZIP 694 kb)

Additional file 2: ADL; Activity of Daily Life-score as a functional questionnaire score. (DOCX 62 kb)

Additional file 3: Clayton; Clayton-100point-score for wrist function. (DOC 56 kb)

Additional file 4: Additional statistic results; relation between different issues and results with statistical analysis. (DOC 289 kb)

## Abbreviations

APW: Anatomical physiological wrist-prosthesis
ATS: Articulo-teno-synovectomy
CHI: Carpal-High-index
ECU: Extensor carpi ulnaris tendon
FFbH: Funktions-Fragebogen Hannover
LDE - Classification: Larsen, Dale and Eek – classification
MPW: Modular physiological wrist-prosthesis
PROMs: Patient reported outcome measures
RIAP: Resection-interposition-arthroplasty
