# Supplementary material for: Radiocarpal fusion and midcarpal resection interposition arthroplasty: long-term results in severely destroyed rheumatoid wrists
Source: BMC Musculoskelet Disord. 2018 Aug 14;19:286. doi: 10.1186/s12891-018-2172-x (PMC6090583; doi:10.1186/s12891-018-2172-x)
Supplement: Supplementary file 1 — Surgical procedure; operation technique with pictures of intraoperative steps and postoperative treatment (4 pictures). (ZIP 694 kb) [file 12891_2018_2172_MOESM1_ESM.zip › Additional file 1.docx]

**Surgical procedure :**

The operation is performed under general anesthesia or regional block using a tourniquet. The patient is placed in the supine position. The incision can be S-shaped transversely or longitudinally on the dorsal side of the wrist. When exposing the wrist, the dorsocarpal retinaculum is incised from the ulnar side, preparing a flap for subsequent fixation of the extensor carpi ulnaris (ECU) tendon. Beginning over the distal ulna, the extensor compartment is entered laterally along the underside of the retinaculum into each compartment, and the retinaculum is then placed like an open book on its radial insertion. The extensor pollicis longus (EPL) tendon is freed from the underside of the dorsocarpal ligament and is moved into a bed of healthy fat along the lateral side of the radius to prevent chafing at Lister's tubercle. Denervation of the posterior interosseous nerve is performed by resection of approximately 1 cm of the nerve. Synovectomy of the extensor tendons is performed, and in cases of ulnar instability, an excision of the distal 2 cm of the ulna is performed. To avoid pain on pronation and supination, the ulnar stump is refixed at the palmar capsule. The arthrodesis of the proximal row (radiolunate and radioscaphoid) is achieved using staples [5, 14], cannulated screws or angular stable plates. Subsequently, approximately 5 mm of the destroyed articular surface of capitate and hamate is resected to rebuild the articular line (fig. 1). A flap of the dorsal capsule or extensor retinaculum is prepared and fixed by interpositioning in the proximal row (fig. 2) [5, 18].

Ligament balancing is easier this way for destructive rheumatoid wrists than for carpal arthrodesis, as in four-corner-fusion.

The extensor carpi ulnaris tendon is captured by Swanson’s slope (special prepared retinacula flap) on top of the dorsal ulna [2] or by distal ulna stabilization. Postoperative a special Vainio finger bandage (fig. 3) and a volar plaster slab for the wrist should be worn for 6 nights [19].

Active isometric finger mobilization and passive careful movements in a range of 30° of flexion and extension, as well as ergotherapy, support the operative result from the first day onward (fig. 4).

*Fig. (1): Resecting the destroyed articular capitate and hamate of approximately 5 mm and rebuilding the articular line.*

*Fig. (2): Fixation of the dorsal capsule as an interposition graft in the proximal row.*

*Fig. (3): Vainio bandage*

*Fig. (4): Postoperative X-ray of the wrist after proximal fusion and distal RIAP.*
