# Supplementary figures and images for: Radiocarpal fusion and midcarpal resection interposition arthroplasty: long-term results in severely destroyed rheumatoid wrists
Source: BMC Musculoskelet Disord. 2018 Aug 14;19:286. doi: 10.1186/s12891-018-2172-x (PMC6090583; doi:10.1186/s12891-018-2172-x)

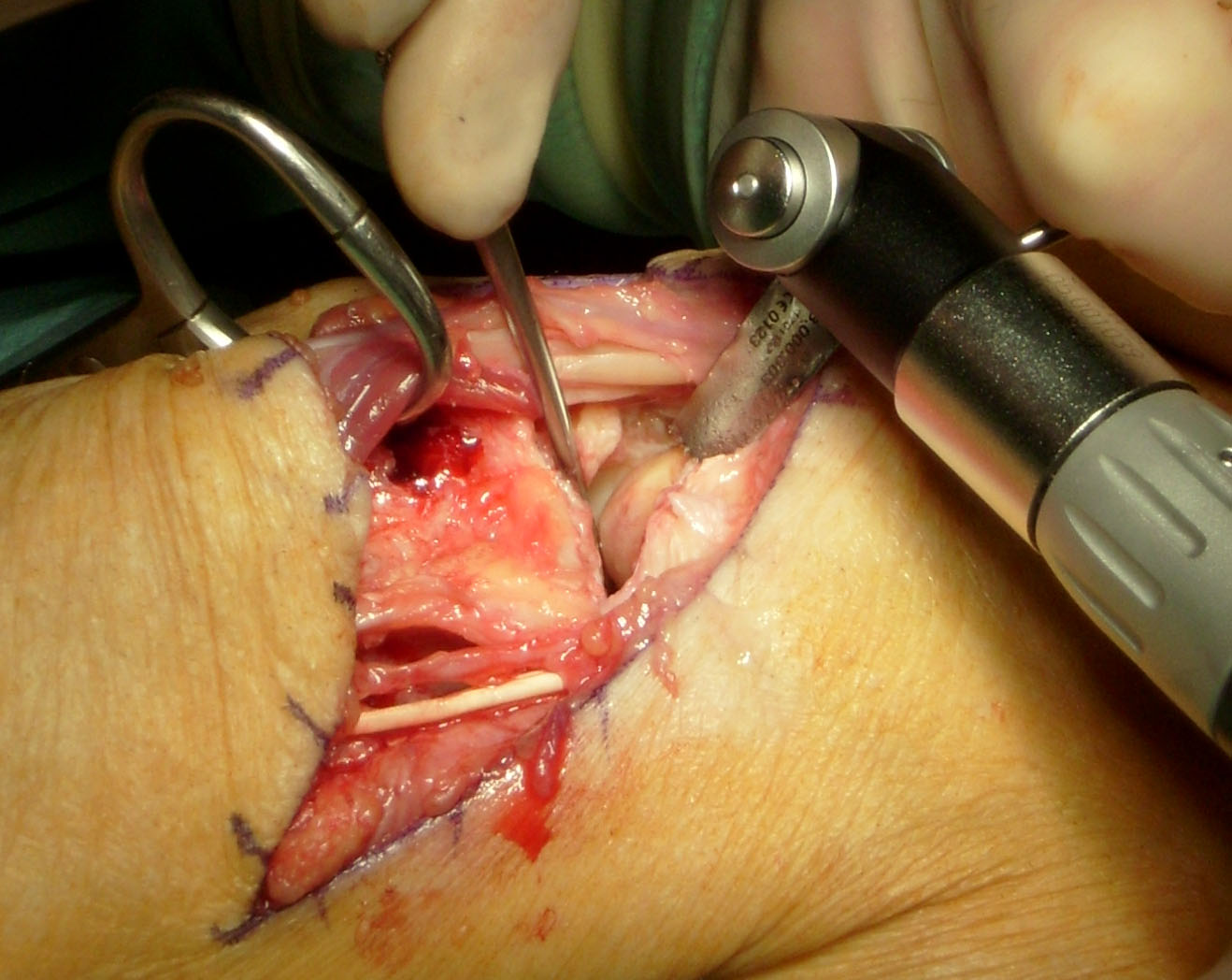

Supplement: Supplementary file 1 — Surgical procedure; operation technique with pictures of intraoperative steps and postoperative treatment (4 pictures). (ZIP 694 kb) [file 12891_2018_2172_MOESM1_ESM.zip › Suppl.Fig.1R6.JPG]

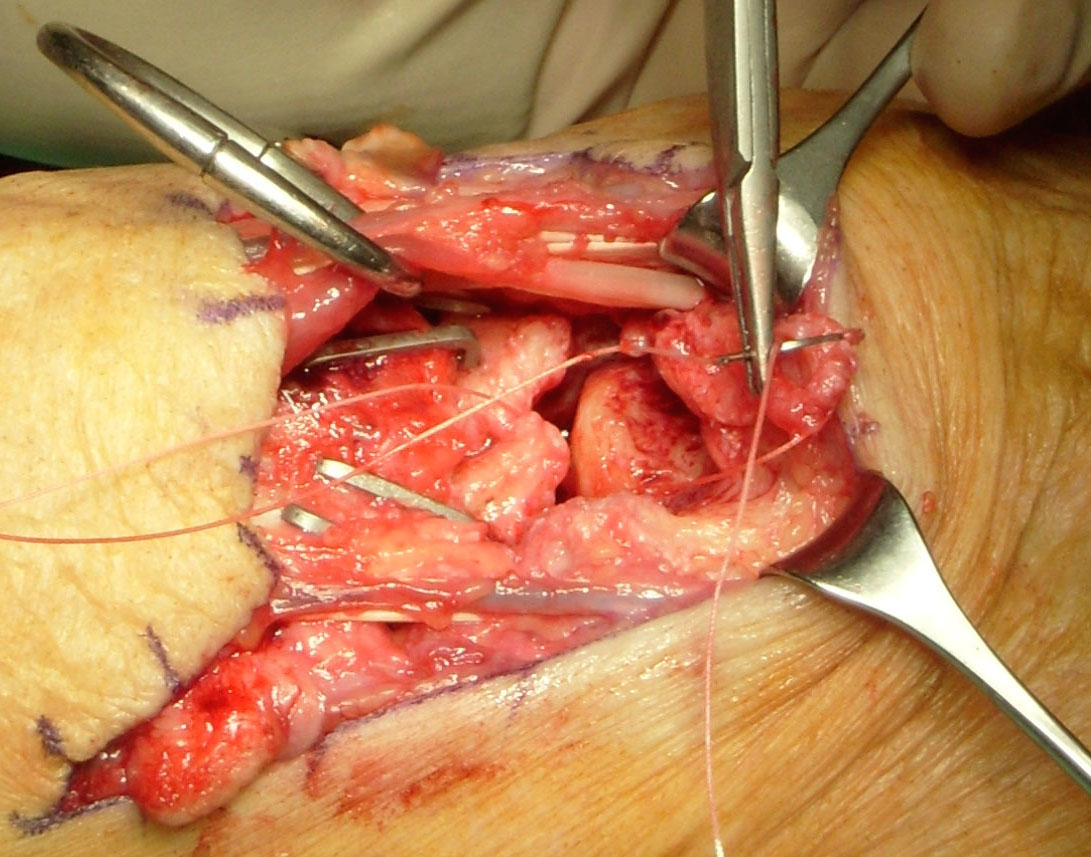

Supplement: Supplementary file 1 — Surgical procedure; operation technique with pictures of intraoperative steps and postoperative treatment (4 pictures). (ZIP 694 kb) [file 12891_2018_2172_MOESM1_ESM.zip › Suppl.Fig.2R6.JPG]

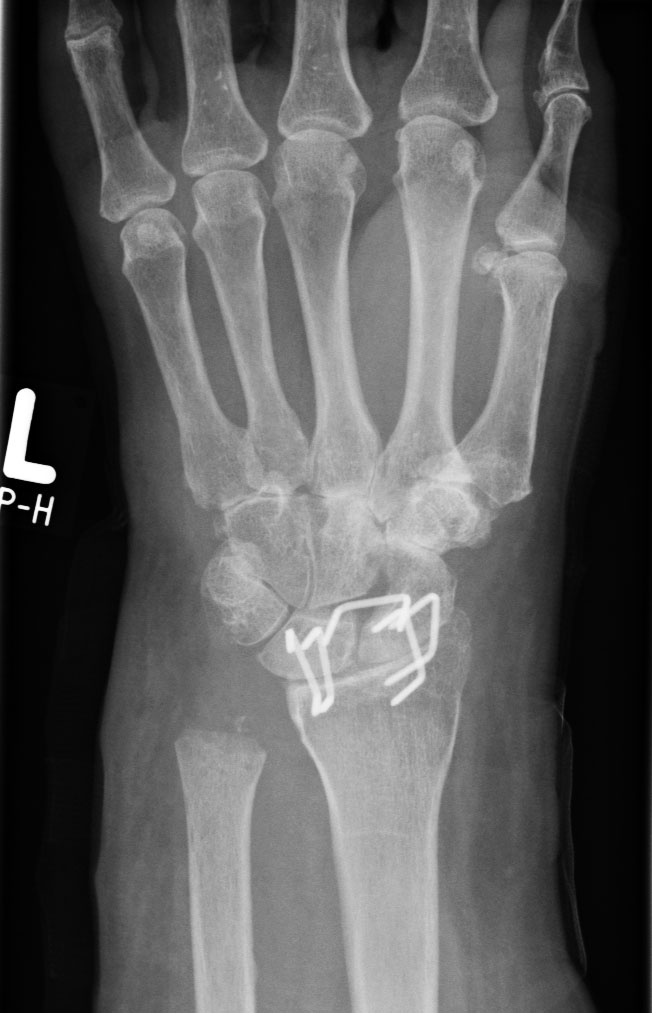

Supplement: Supplementary file 1 — Surgical procedure; operation technique with pictures of intraoperative steps and postoperative treatment (4 pictures). (ZIP 694 kb) [file 12891_2018_2172_MOESM1_ESM.zip › Suppl.Fig.4R6.jpg]

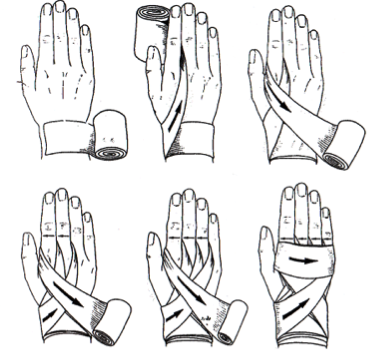

Supplement: Supplementary file 1 — Surgical procedure; operation technique with pictures of intraoperative steps and postoperative treatment (4 pictures). (ZIP 694 kb) [file 12891_2018_2172_MOESM1_ESM.zip › Suppl.Fig.3R6.png]
