# Supplementary material for: Radiocarpal fusion and midcarpal resection interposition arthroplasty: long-term results in severely destroyed rheumatoid wrists
Source: BMC Musculoskelet Disord. 2018 Aug 14;19:286. doi: 10.1186/s12891-018-2172-x (PMC6090583; doi:10.1186/s12891-018-2172-x)
Supplement: Supplementary file 2 — ADL; Activity of Daily Life-score as a functional questionnaire score. (DOCX 62 kb) [file 12891_2018_2172_MOESM2_ESM.docx]

10 Questions are used to access the daily activites (40 points):

- - Using the knife and fork
  - Hygiene
  - Hair styling
  - Scissors using
  - Elevation of the hat
  - To pick up coins
  - Writing a card
  - Lay down over the hand
  - Using the keys
  - Opening a bottle

For each question the patient has to choose one answer:

| - none = 4 |
| --- |
| - slightly = 3 |
| - moderate = 2 |
| - severe = 1 |

**Results:**

40-30 pts. = normal

30-20 pts. = mild affection

20-10 pts. = moderate

< 10 severely affected

**Patient satisfaction**

- Visual analogue scale ( 0 – 10 pts.)
- Satisfaction with the operation

(do you accept another operation like this again?)

- Pain after operation (none, slightly, moderate, severe)
