# Supplementary material for: Radiocarpal fusion and midcarpal resection interposition arthroplasty: long-term results in severely destroyed rheumatoid wrists
Source: BMC Musculoskelet Disord. 2018 Aug 14;19:286. doi: 10.1186/s12891-018-2172-x (PMC6090583; doi:10.1186/s12891-018-2172-x)
Supplement: Supplementary file 3 — Clayton; Clayton-100point-score for wrist function. (DOC 56 kb) [file 12891_2018_2172_MOESM3_ESM.doc]

| **Parameter** | **Score** |
| --- | --- |
| 1. **Balance: 30 points** 2. Active flexion minus extension (Positive Value): 15 points   Excellent : 0-20  Good: 21-30  Fair : 31-40  Poor: >40  B. Active ulnar deviation minus radial deviation (Positive value): 15 points  Excellent : 0-20  Good: 21-30  Fair : 31-40  Poor: >40 | 15  10  5  0  15  10  5  0 |
| 1. **Rang of motion (Sum of flexion and extension, in °): 15 points**   Excellent : 70-90  Good: 50-69  Fair : 15-49  Poor: <15 | 15  10  5  0 |
| 1. **Pain relief: 35 points**   No pain  Mild  Moderate  Severe | 35  30  7  0 |
| 1. **Wrist extensor strength (Pounds): 10 points**   >5  2  1  <1 | 10  6  3  0 |
| 5**. Patient satisfaction: 10 points**  Yes  No | 10  0 |
| **Total Score** | 100 |

**Results:**  90-100 pts = excellent

70-89 pts = good

60-69 pts = satisfied

<60 pts = poor
