# Supplementary material for: Radiocarpal fusion and midcarpal resection interposition arthroplasty: long-term results in severely destroyed rheumatoid wrists
Source: BMC Musculoskelet Disord. 2018 Aug 14;19:286. doi: 10.1186/s12891-018-2172-x (PMC6090583; doi:10.1186/s12891-018-2172-x)
Supplement: Supplementary file 4 — Additional statistic results; relation between different issues and results with statistical analysis. (DOC 289 kb) [file 12891_2018_2172_MOESM4_ESM.doc]

**Table S1: The relation between duration of the disease and the final results.**

|  |  | **Minimum** | **Maximum** | **Mean** | **Std. Deviation** | **t** | **p** |
| --- | --- | --- | --- | --- | --- | --- | --- |
| **Duration of the disease** | **Satisfactory** | 10.00 | 33.00 | 16.4167 | 6.27567 | 2.058 | .026 |
|  | **Unsatisfactory** | 9.00 | 54.00 | 22.4000 | 12.92027 |  |  |

**Table S2: Relation between the duration of the disease before the operation**

|  |  | **Minimum** | **Maximum** | **Mean** | **Std. Deviation** | **t** | **p** |
| --- | --- | --- | --- | --- | --- | --- | --- |
|  |  |  |  |  |  |  |  |
| **Time gap (month)** | **Satisfactory** | .00 | 292.00 | 94.2083 | 66.65615 | 1.962 | .049* |
|  | **Unsatisfactory** | -2.00 | 373.00 | 147.0000 | 110.73291 |  |  |
|  |  |  |  |  |  |  |  |

Fig. S1 Correlation between duration of the disease and Clayton score.

**Fig. S2) The Correlation between the time gap between the beginning of the disease and operation**

**Table S3**: Pain score in relation to the final results:

|  |  | **Mean** | **Std. Deviation** | **Minimum** | **Maximum** | **t** | **Sig.** |
| --- | --- | --- | --- | --- | --- | --- | --- |
| **Pain Score** | **Satisfactory** | 3.0000 | 1.47710 | .00 | 5.00 | 4.035 | .058 |
|  | **Unsatisfactory** | 4.8000 | 2.65832 | 1.00 | 8.00 |  |  |

**Table S4**: Pain in relation to Clayton final score

| Pain | |  | Clayton final score | | Total |
| --- | --- | --- | --- | --- | --- |
|  |  |  | Satisfactory | Unsatisfactory |  |
|  | No | No | 13 | 0 | 13 |
|  |  | % | 54.2% | .0% | 38.2% |
|  | Mild | No | 5 | 3 | 8 |
|  |  | % | 20.8% | 30.0% | 23.5% |
|  | Moderate | No | 6 | 4 | 10 |
|  |  | % | 25.0% | 40.0% | 29.4% |
|  | Severe | No | 0 | 3 | 3 |
|  |  | % | .0% | 30.0% | 8.8% |
| Total | | No | 24 | 10 | 34 |
|  | | % | 100.0% | 100.0% | 100.0% |
| X^2^  p | |  | 13.409  .004* | |  |

**Table S5: the relation between the swelling and the power after the operation**

|  | |  | **Power after the operation** | | | | **Total** |
| --- | --- | --- | --- | --- | --- | --- | --- |
|  |  |  | **Better** | **No difference** | **Worse** | **Very bad** |  |
| **Swelling** | **No - Mild** | **No.** | 22 | 5 | 3 | 2 | 32 |
|  |  | **%** | 100.0% | 100.0% | 100.0% | 50.0% | 94.1% |
|  | **Moderate - severe** | **No.** | 0 | 0 | 0 | 2 | 2 |
|  |  | **%** | .0% | .0% | .0% | 50.0% | 5.9% |
| **Total** | | **No.** | 22 | 5 | 3 | 4 | 34 |
|  |  | **%** | 100.0% | 100.0% | 100.0% | 100.0% | 100.0% |
| **X^2^**  **p** | |  | 15.937  .001 | | | |  |

**Table S6: Relation between range of motion and final results.**

| Range of motion  Result | Flexion/Extension | Abd-/Adduction | Pro-/supination |
| --- | --- | --- | --- |
| Satisfactory | 15.97-0-22.5 | 7.2-0-13 | 73.75-0-47.9 |
| Unsatisfactory | 11-0-16.6 | 4.5-0-8.7 | 63-0-43.5 |

**Table S7**: Relation between range of motion and final results.

|  |  | **Minimum** | **Maximum** | **Mean** | **Std. Deviation** | **t** | **p** |
| --- | --- | --- | --- | --- | --- | --- | --- |
| **Flexion** | **Satisfactory** | .00 | 50.00 | 15.9792 | 12.08873 | 1.09 | .254 |
|  | **Unsatisfactory** | .00 | 25.00 | 11.0000 | 9.36898 |  |  |
|  |  |  |  |  |  |  |  |
| **Extension** | **Satisfactory** | .00 | 55.00 | 22.5000 | 13.92214 | 1.163 | .289 |
|  | **Unsatisfactory** | .00 | 41.00 | 16.6000 | 15.98750 |  |  |
|  |  |  |  |  |  |  |  |
| **Ulnar** | **Satisfactory** | .00 | 40.00 | 13.0417 | 10.14023 | 0.877 | .286 |
|  | **Unsatisfactory** | .00 | 35.00 | 8.7000 | 11.79501 |  |  |
|  |  |  |  |  |  |  |  |
| **Radial** | **Satisfactory** | .00 | 25.00 | 7.2083 | 6.73072 | 1.059 | .311 |
|  | **Unsatisfactory** | .00 | 20.00 | 4.5000 | 7.61942 |  |  |
|  |  |  |  |  |  |  |  |
| **Pronation** | **Satisfactory** | .00 | 90.00 | 73.7500 | 23.18405 | 1.156 | .290 |
|  | **Unsatisfactory** | .00 | 90.00 | 63.0000 | 33.68151 |  |  |
|  |  |  |  |  |  |  |  |
| **Supination** | **Satisfactory** | .00 | 90.00 | 47.9167 | 24.97462 | .217 | .645 |
|  | **Unsatisfactory** | .00 | 80.00 | 43.5000 | 25.82527 |  |  |
|  |  |  |  |  |  |  |  |

**Table S8: The relation the balance in the sagittal plane and** Clayton score

|  | |  | Clayton score final | | Total |
| --- | --- | --- | --- | --- | --- |
|  | |  | Satisfactory | Unsatisfactory |  |
| Balance Sagital | Excellent | No. | 23 | 9 | 32 |
|  |  | % | 95.8% | 90% | 94.1% |
|  | good | No. | 1 | 0 | 1 |
|  |  | % | 4.2% | .0% | 2.9% |
|  | bad | No. | 0 | 1 | 1 |
|  |  | % | .0% | 10% | 2.9% |
| Very bad  Total | | No  %  No. | 0  0  24 | 0  0  10 | 0  0  34 |
|  | | % | 100.0% | 100.0% | 100.0% |
| X^2^  p | |  | 2.842  .241 | |  |

**Table S9: Balance frontal and** Clayton score

|  | |  | Clayton score final | | Total |
| --- | --- | --- | --- | --- | --- |
|  | |  | Satisfactory | Unsatisfactory |  |
| Balance Frontal | Excellent | No. | 20 | 8 | 28 |
|  |  | % | 83.3% | 80% | 82.4% |
|  | Good | No. | 4 | 1 | 5 |
|  |  | % | 16.7% | 10.0% | 14.7% |
|  | Bad | No. | 0 | 1 | 1 |
|  | Very Bad | %  No  % | .0%  0  0 | 6.7%  0  0 | 2.9%  0  0 |
| Total | | No. | 24 | 10 | 34 |
|  | | % | 100.0% | 100.0% | 100.0% |
| X^2^  p | |  | 2.623  .269 | |  |

**Table S10**: Power after the operation in relation to Clayton score final

| Power after the operation | |  | Clayton score final | | Total |
| --- | --- | --- | --- | --- | --- |
|  | |  | Satisfactory | Unsatisfactory |  |
|  | Better | No. | 18 | 4 | 22 |
|  |  | % | 75% | 40% | 64.7% |
|  | No difference | No. | 3 | 2 | 5 |
|  |  | % | 12.5% | 20% | 14.7% |
|  | Worse | No. | 2 | 1 | 3 |
|  |  | % | 8.3% | 10% | 8.8% |
|  | Very bad | No. | 1 | 3 | 4 |
|  |  | % | 4.2% | 30% | 11.8% |
| Total | | No. | 24 | 10 | 34 |
|  | | % | 100.0% | 100.0% | 100.0% |
| X^2^  p | |  | 5.633  .028 | |  |

**Table S11**: Relation between Wrist grip strength and Clayton score:

|  |  | **Minimum** | **Maximum** | **Mean** | **Std. Deviation** | **t** | **p** |
| --- | --- | --- | --- | --- | --- | --- | --- |
|  |  |  |  |  |  |  |  |
| **Weight** | **Satisfactory** | .00 | 15.00 | 6.6458 | 4.47816 | 2.619 | .024* |
|  | **Unsatisfactory** | .00 | 8.00 | 2.9900 | 2.90610 |  |  |
|  |  |  |  |  |  |  |  |
| **Grip average** | **Satisfactory** | .05 | .65 | .2833 | .12426 | 2.935 | .016* |
|  | **Unsatisfactory** | .00 | .38 | .1690 | .10847 |  |  |
|  |  |  |  |  |  |  |  |
| **Key pinch** | **Satisfactory** | .15 | .30 | .2083 | .05845 | 1.935 | .045* |
|  | **Unsatisfactory** | .35 | .35 | .3500 | . |  |  |
|  |  |  |  |  |  |  |  |
| **Tip pinch** | **Satisfactory** | .15 | .40 | .2583 | .11583 | .537 | .497 |
|  | **Unsatisfactory** | .35 | .35 | .3500 | . |  |  |
|  |  |  |  |  |  |  |  |

**Fig. S3: Carrying capacity after the operation in relation to Clayton score final**

**Table S12: Wrist extension strength in relation to Clayton final score**

|  | |  | **Clayton final score** | | **Total** |
| --- | --- | --- | --- | --- | --- |
|  |  |  | **Satisfactory** | **Unsatisfactory** |  |
| **Wrist extension strength** | **Very good** | **No.** | 18 | 2 | 20 |
|  |  | **%** | 75.0% | 20.0% | 58.8% |
|  | **Good** | **No.** | 3 | 2 | 5 |
|  |  | **%** | 12.5% | 20.0% | 14.7% |
|  | **Fair** | **No.** | 3 | 5 | 8 |
|  |  | **%** | 12.5% | 50.0% | 23.5% |
|  | **Bad** | **No.** | 0 | 1 | 1 |
|  |  | **%** | .0% | 10.0% | 2.9% |
| **Total** | | **No.** | 24 | 10 | 34 |
|  |  | **%** | 100.0% | 100.0% | 100.0% |
| **X^2^**  **p** | |  | 10.519  .015 | |  |

**Table S13: Finger problems in relation to power after the operation**

|  | |  | **Power after the operation** | | | | **Total** |
| --- | --- | --- | --- | --- | --- | --- | --- |
|  |  |  | **Better** | **No difference** | **Worse** | **Very bad** |  |
| **Finger** | **No - Mild** | **No.** | 15 | 1 | 2 | 0 | 18 |
|  |  | **%** | 68.2% | 20.0% | 66.7% | .0% | 52.9% |
|  | **Moderate - severe** | **No.** | 7 | 4 | 1 | 4 | 16 |
|  |  | **%** | 31.8% | 80.0% | 33.3% | 100.0% | 47.1% |
| **Total** | | **No.** | 22 | 5 | 3 | 4 | 34 |
|  |  | **%** | 100.0% | 100.0% | 100.0% | 100.0% | 100.0% |
| **X^2^**  **p** | |  | 8.956  .030 | | | |  |

**Table S14**: Shoulder problems in relation to power after the operation

|  | |  | **Power after the operation** | | | | **Total** |
| --- | --- | --- | --- | --- | --- | --- | --- |
|  |  |  | **Better** | **No difference** | **Worse** | **Very bad** |  |
| **Shoulder** | **No - Mild** | **No.** | 15 | 5 | 1 | 3 | 24 |
|  |  | **%** | 68.2% | 100.0% | 33.3% | 75.0% | 70.6% |
|  | **Moderate - severe** | **No.** | 7 | 0 | 2 | 1 | 10 |
|  |  | **%** | 31.8% | .0% | 66.7% | 25.0% | 29.4% |
| **Total** | | **No.** | 22 | 5 | 3 | 4 | 34 |
|  |  | **%** | 100.0% | 100.0% | 100.0% | 100.0% | 100.0% |
| **X^2^**  **p** | |  | 4.188  .242 | | | |  |

**Table S15**: Shoulder problems in relation to Clayton score final

|  | |  | Clayton score final | | Total |
| --- | --- | --- | --- | --- | --- |
|  | |  | Satisfactory | Unsatisfactory |  |
| Shoulder | No - Mild | No. | 17 | 7 | 24 |
|  |  | % | 70.8% | 70% | 70.6% |
|  | Moderate - severe | No. | 7 | 3 | 10 |
|  |  | % | 29.2% | 30% | 29.4% |
| Total | | No. | 24 | 10 | 34 |
|  | | % | 100.0% | 100.0% | 100.0% |
| X^2^  p | |  | .961  .002 | |  |

**Table S16: Elbow problems in relation to power after the operation**

|  | |  | **Power after the operation** | | | | **Total** |
| --- | --- | --- | --- | --- | --- | --- | --- |
|  |  |  | **Better** | **No difference** | **Worse** | **Very bad** |  |
| **Elbow** | **No - Mild** | **No.** | 17 | 5 | 1 | 3 | 26 |
|  |  | **%** | 77.3% | 100.0% | 33.3% | 75.0% | 76.5% |
|  | **Moderate - severe** | **No.** | 5 | 0 | 2 | 1 | 8 |
|  |  | **%** | 22.7% | .0% | 66.7% | 25.0% | 23.5% |
| **Total** | | **No.** | 22 | 5 | 3 | 4 | 34 |
|  |  | **%** | 100.0% | 100.0% | 100.0% | 100.0% | 100.0% |
| **X^2^**  **p** | |  | 4.654  .199 | | | |  |

**Table S17**:Elbow problems in relation to Clayton score final

|  | |  | Clayton score final | | Total |
| --- | --- | --- | --- | --- | --- |
|  | |  | Satisfactory | Unsatisfactory |  |
| Elbow | No - Mild | No. | 19 | 7 | 26 |
|  |  | % | 79.2% | 70% | 76.5% |
|  | Moderate - severe | No. | 5 | 3 | 8 |
|  |  | % | 20.8% | 30% | 23.5% |
| Total | | No. | 24 | 10 | 34 |
|  | | % | 100.0% | 100.0% | 100.0% |
| X^2^  p | |  | .330  .566 | |  |

**Table S18**:Finger problems in relation to Clayton score final

|  | |  | Clayton score final | | Total |
| --- | --- | --- | --- | --- | --- |
|  | |  | Satisfactory | Unsatisfactory |  |
| Finger | No - Mild | No. | 15 | 3 | 18 |
|  |  | % | 62.5% | 30% | 52.9% |
|  | Moderate - severe | No. | 9 | 7 | 16 |
|  |  | % | 37.5% | 70% | 47.1% |
| Total | | No. | 24 | 10 | 34 |
|  | | % | 100.0% | 100.0% | 100.0% |
| X^2^  p | |  | 2.993  .084 | |  |

**Table S19: the relation between the CHI, UTI with final results:**

|  | Clayton score final | N | Min. | Max. | Mean | S.D. | F | Sig. |
| --- | --- | --- | --- | --- | --- | --- | --- | --- |
|  |  |  |  |  |  |  |  |  |
| Carpus height index | Satisfactory | 19 | 0.3 | 0.58 | 0.40 | 0.08 | .163 | .689 |
|  | Unsatisfactory | 15 | 0.2 | 0.5 | 0.39 | 0.07 |  |  |
|  |  |  |  |  |  |  |  |  |
| Ulnar translation of the carpus | Satisfactory | 19 | 0.2 | 0.35 | 0.28 | 0.04 | 1.258 | .270 |
|  | Unsatisfactory | 15 | 0.16 | 0.36 | 0.26 | 0.06 |  |  |
